# Publication trends of somatic mutation and recombination tests research: a bibliometric analysis (1984‒2020)

**DOI:** 10.5808/gi.21083

**Published:** 2022-03-31

**Authors:** Ghada Tagorti, Bülent Kaya

**Affiliations:** Department of Biology, Faculty of Sciences, Akdeniz University, 07058 Campus, Antalya, Turkey

**Keywords:** bibliometric, cancer, *Drosophila*, genotoxicity, SMART, Web of Science

## Abstract

Human exposure to pollutants has been on the rise. Thus, researchers have been focused on understanding the effect of these compounds on human health, especially on the genetic information by using various tests, among them the somatic mutation and recombination tests (SMARTs). It is a sensitive and accurate method applicable to genotoxicity analysis. Here, a comprehensive bibliometric analysis of SMART assays in genotoxicity studies was performed to assess publication trends of this field. Data were extracted from the Web of Science database and analyzed by the bibliometric tools HistCite, Biblioshiny (RStudio), VOSViewer, and CiteSpace. Results have shown an increase in the last 10 years in terms of publication. A total of 392 records were published in 96 sources mainly from Brazil, Spain, and Turkey. Research collaboration networks between countries and authors were performed. Based on document co-citation, five large research clusters were identified and analyzed. The youngest research frontier emphasized on nanoparticles. With this study, how research trends evolve over years was demonstrated. Thus, international collaboration could be enhanced, and a promising field could be developed.

## Introduction

Anthropogenic activities disseminate large amounts of chemical substances into the environment [1]. Hence, humans are currently exposed to several pollutants with genotoxic potentials such as metals [2], pesticides [3], industrial waste mix [4], and nanomaterials [5]. Genotoxicity is a wide term comprising DNA damage and mutagenicity, where the mutagenic effect is described as an occurred event with irreversible and heritable outcomes affecting the DNA and/or chromosome structure [6]. The genotoxic effect recorded on somatic cells has been associated with pathological endpoints such as premature aging, neuronal diseases, and even carcinogenesis [7,8]. Accordingly, the genotoxicity evaluation is a required component in the human health risk assessment and as one single test is unable to detect all the genotoxic endpoints, a battery of *in vitro* and *in vivo* tests was recommended [9]. Among these assays, the somatic mutation and recombination tests (SMARTs) are one of the commonly used tests. The SMART assays are *in vivo* assay to assess the potential genotoxicity of substances in the somatic cells of *Drosophila melanogaster* [10]. This assay could target wing cells named as wing-spot test proposed first time by Graf et al. [11], or eyes cells known as eye-spot test defined by Wurgler and Vogel [12]. In both cases, losing heterozygosity by deletions, point mutations, mitotic recombination, and nondisjunction unravels the expression of genetic markers in heterozygous or transheterozygous individuals, ensuring the quantification of the damage by visual scoring [13]. The wing-spot test includes two types of cross using recessive genetic markers on the 3rd chromosome, a standard cross with normal bioactivation between female virgins (*flr^3^/In (3LR)TM3, ri p^p^sep l(3)89Aa bx^34e^ e Bd^s^*) and (*mwh/mwh*) males, and a high metabolic bioactivation cross with high levels of cytochrome P450 between female virgins (ORR; *flr^3^/In (3LR)TM3, ri p^p^sep l(3)89Aa bx^34e^ e Bd^s^*) and (*mwh/mwh*) males. The mutant spots are produced after chemical exposure that induced point mutation, deletion, or mitotic recombination [14,15]. The eye-spot test is based on a cross between wild-type eyed females (*w*+/*w*+) and white-eyed males (*w*/Y). The gene white (*w*) is a recessive marker found on the X chromosome. During the offspring period, a mutagenic event could occur and cause the formation of white phenotype spots (mutant ommatidia) in the wild-type eyes [16]. Although both tests are accurate, sensitive, and specific, the wing-spot test allows the visual scoring of wings over time, whereas in the eye-spot test, the analysis should be performed quickly since no preserving actions are available on the eyes [17]. In fact, the SMART assays have been applied in the analysis of the genotoxicity and the antimutagenicity of several chemicals and agents such as pesticides [18-20], nanomaterials [21,22], food products [23,24], hormones [25], plant extracts [26], and drugs [27-29]. With this variety of studies, a bibliometric analysis is required to assess the impact of this methodology on the genotoxicity studies. Bibliometric analysis is considered a highly sensitive method to evaluate research outputs based on statistical tools and to study metrological features of information created in a specific field [30]. To the best of our knowledge, no paper using the bibliometric analysis to explore the trends of SMART assays research has been published. Therefore, in the current study, various aspects were exanimated to evaluate the publications and citation trends in SMART assays from 1984 to 2020. Hence, the following research objectives were considered guiding the study design: (1) to identify the most influential journals and publications, the impactful authors and institutions, and the leading countries in SMARTs literature; (2) to find the patterns of collaboration between countries and authors within this research domain; (3) to explore the emerging keywords and research themes. The findings of the present study will provide a comprehensive overview of the importance of SMART assays, it would provide information to the scholars to easily identify the research profile and enhance collaboration.

## Methods

**Fig. 1.**
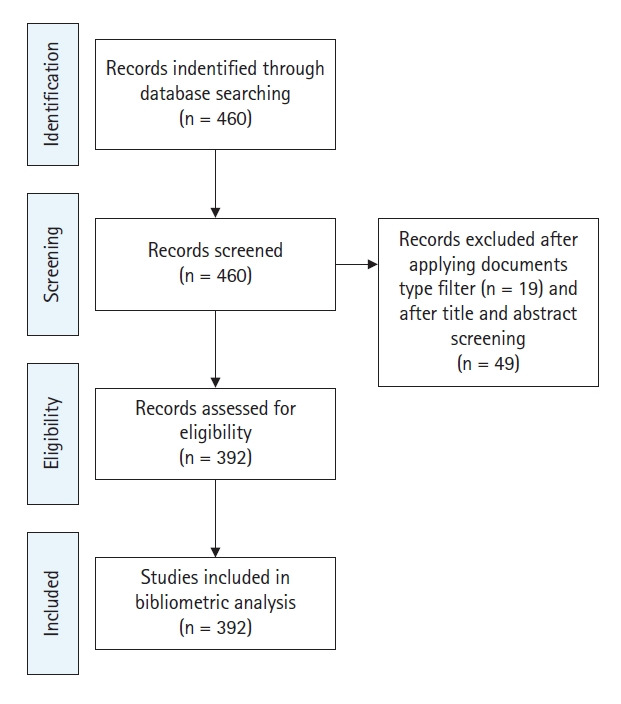
PRISMA flow diagram.

This bibliometric study analyzed the published academic studies of SMARTs indexed in the Web of Science (WOS) core collection database. As known, WOS is the most reliable global citation database with a collection of over 21,000 peer-reviewed journals and the most accepted one for analysis of academic papers [31]. A comprehensive four-step approach was framed in this study as shown in Fig. 1. Boolean operators were used with adding all the relevant keywords to retrieve more relevant papers. (TS = ((“SMART assays” OR “SMART assay” OR “SMART test” OR “SMART tests” OR “wing-spot test” OR “wing-spot assay” OR “somatic mutation and recombination test” OR “eye-spot test” OR “eye-spot assay” OR “small single spots” OR “large single spots” OR “mutant ommatidia” OR “mutant eye unit” OR “w/*w*+ SMART”))). The search was performed on 14 January 2021; it is essential to present the date of records collection as the database is constantly updating [32]. Most bibliometric studies are based on academic articles [30], thereby this study was limited to original articles written in English in the strict sense. The search was also limited to 2020. The authors adopted the PRISMA approach, which has been used in bibliometric studies. A total of 460 records were extracted initially, which later were filtered by document types, to exclude the following document types: proceedings papers, book chapters, and early access. The data relevance and accuracy were assured by scanning the title and abstract of each record, and 49 records were excluded. The irrelevant articles were from the following categories: (1) Internet of things and wireless sensing, (2) communication services, (3) image processing, (4) industry, and (5) single molecule amplification and re-sequencing technology (SMART). Finally, a total of 392 records were remained to be downloaded in plain text to extract the following data: publication year, author, title, abstract, keywords, cited references, journal title, and institution, for further analysis.

Having selected 392 relevant records, data visualization and analysis were conducted by using various bibliometric software and applications, including Microsoft Excel, HistCite, Biblioshiny (RStudio), VOSViewer, and CiteSpace. The analysis was performed in three stages. First, a bibliometric citation analysis was performed (number of publications and citations, relevant journals, productive institutions, and the most impactful articles and authors). Secondly, a network analysis was applied, including collaboration between countries and co-authoring using the Walktrap clustering algorithm. This algorithm has the advantage to be computed effectively and placing the data in a network [33]. Furthermore, a three-fields-plot based on the Sankey diagram to indicate the interrelation between keywords, journals, and countries was visualized. Detection of the emerging research fields is required to outline research areas. Therefore, thirdly, a content analysis was conducted. HistCite (version 12.03.17) was used for the bibliometric citation analysis to sort the collected data by quantitative (number of publications and citations) and qualitative indicators (total global citation score and total local global citation score). Hence, these two types of indicators were applied in the current study. The total global citation score (TGCS) represents the number of citations of a paper included in the collection selected for the analysis in the WOS whereas the total local citation score (TLCS) refers to the total number of citations of a paper included in the collection and has been cited by other papers of the same collection [34]. The RStudio software (version 1.3.1093) with Biblioshiny application was used [35]. Hence, this application was also used to analyze the basic indicators of the search, plus the collaboration between countries and authors, and the three-fields plot. VOSViewer software (version 1.6.16) was used to analyze and visualize the emerging keywords. CiteSpace (version 5.7.R4) was used to perform the co-citation analysis and to investigate the emerging topics. Co-citation analysis is an effective tool to understand the intellectual structure of a research field, with the cited papers intellectual bases are revealed whereas papers in their active state of citation represent the research frontiers which usually display characteristics of a field specificity [36]. One of the important tools found in CiteSpace is the betweenness centrality. A paper with high betweenness centrality was defined as an important research paper in the network [37]. Furthermore, articles with strong citation bursts with time slices were identified. The burst is observed when a publication has an excess in its citation counts compared to its peers. This furthers identify publications that interested the scholars over time and thereby help to explore the research frontiers of a given field [38,39].

## Results

The present study analyzed the SMART assays in the genotoxicity studies published during 1984-2020. A total of 392 records have been written by 933 authors from 35 different countries with an average of 17.01 citations per document. Authors of single-authored documents are seven (0.75%) while authors of multi-authored documents represent 926 authors (99.25%).

### Basic indicators

#### Yearly publication and citation

**Fig. 2.**
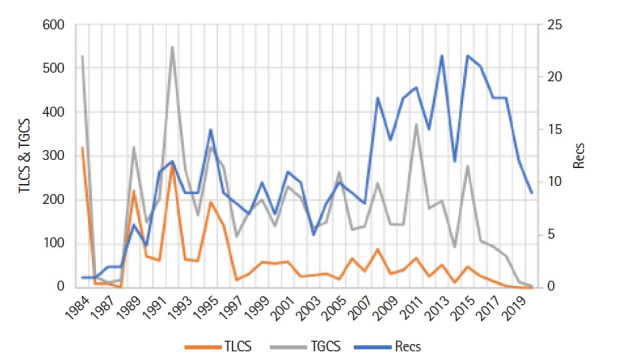
Evolution of the number of articles and citations over the years. Recs, number of publications; TGCS, total global citation score; TLCS, total local citation score.

The first publication appeared in 1984, thereafter the number of publications has been gradually increasing at a rate of 14.4 articles per year with a registered bloom in 2013 and 2015 (Recs, 22), whereas the TLCS and TGCS have recorded the bloom in 1984 (TLCS, 319; TGCS, 527) and in 1992 (TLCS, 282; TGCS, 547). The output of the first 25 years (1984‒2009) was 206 publications and only in the past 10 years (2010‒2020), 186 studies were published. This finding indicates the growing interest in using SMART assays in genotoxicity studies (Fig. 2).

#### Authors

The top 10 authors collectively contributed to 302 studies. Graf U, Marcos R, and de Andrade HHR are the top three authors in SMART assays field (Table 1). Graf U is the most influential author with the highest number of TLCS (953) and TGCS (1,716).

#### Institutions and countries

The top 10 institutions published 70.2% (n = 275) studies of the total publications. The Federal University of Uberlândia in Brazil is on the top of the list with 39 articles (TGCS, 425) (Table 2). Interestingly, the University of Zürich ranked in 4th position has the highest TGCS (1,892) and TLCS (1,119). There is only one country having a number of publications in three-digits, Brazil, with 106 publications and 1,103 TGCS, followed by Spain (TGCS, 1,470; TLCS, 338) and Turkey (TGCS, 784; TLCS, 221).

#### Journals

The 392 studies in SMART assays were published in 96 academic sources. More than half of these studies (n = 241, 61.5%) were published in the top 10 journals. The sources ‘Food and Chemical Toxicology’ and ‘Mutation Research Genetic Toxicology and Environmental Mutagenesis’ are on the top of the list with 53 publications (Table 3). The journal with the highest impact factor Chemosphere (7.086) has published 10 studies with 177 TGCS.

#### Articles

The years of the top 10 highly cited articles ranged from 1984 to 1996. There is only one article that obtained over 200 citations. This article has been titled ‘Somatic Mutation and Recombination Test in *Drosophila melanogaster*’ by Graf et al. and published in 1984 [11]. Half of the highly cited articles were published in Mutation Research journal, and this journal is on the top list of influential journals (Table 4).

### Network Analysis

#### Co-authorship

**Fig. 3.**
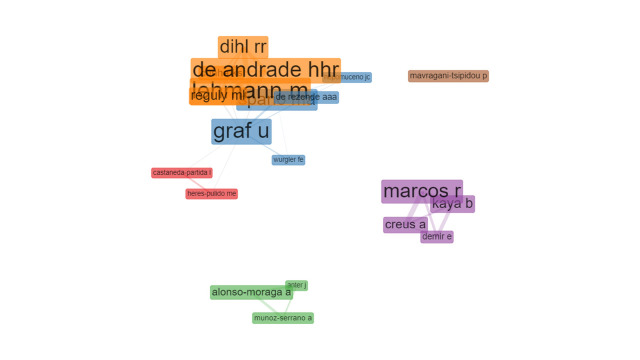
Co-authorship network (Walktrap algorithm, association normalization, 20 nodes, 1 minimum edge, created by Biblioshiny).

Each node represents an author and the edges indicate the research collaboration between them. Six clusters are recorded, the blue and the orange clusters both with five authors are the largest clusters, followed by the purple cluster which includes four authors (Fig. 3). The brown cluster has one separate author.

#### Collaboration between countries

**Fig. 4.**
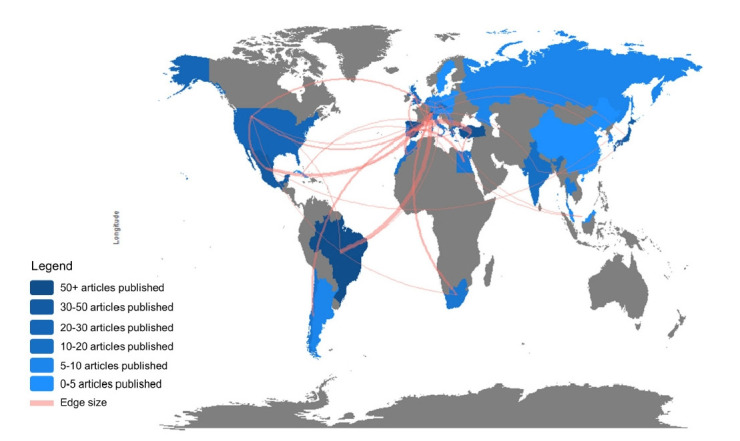
Country collaboration map (created by Biblioshiny).

A total of 38 collaboration entries are registered worldwide. Turkey, Brazil, and Mexico were the most collaborative countries (Fig. 4).

#### Co-word

**Fig. 5.**
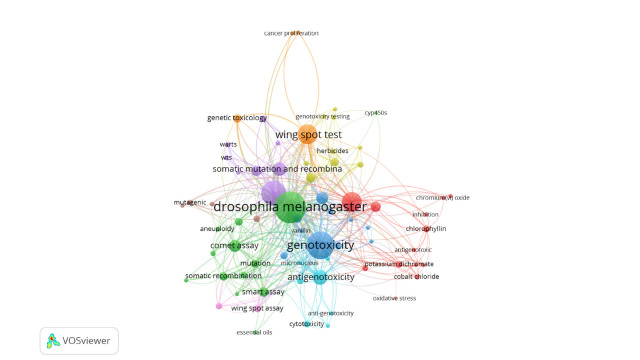
Co-occurrence network of author keywords. The green and the red clusters are the strongest ones (12 words) followed by the blue cluster (10 words). The top word is *Drosophila melanogaster* with the maximum occurrence in somatic mutation and recombination test assays literature.

The co-occurrence of keywords represents the relationship between two words that occurred together. Three minimum number of occurrences of a keyword were selected; hence, out of 861 authors’ keywords, 70 meet this criterion to form 11 clusters (Fig. 5). Each color indicates a separate cluster and clusters are organized based on the link strength and occurrence. Thus, the size of the bubble represents the relationship between link strength and occurrence. The first five keywords with the high total link strength are *Drosophila melanogaster* (link strength: 320), genotoxicity (247), SMART (181), *Drosophila* (108), and wing-spot test (105).

#### Three-fields plot

**Fig. 6.**
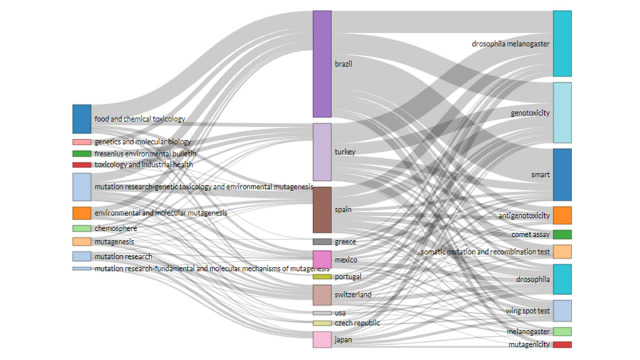
Three-fields plot (created by Biblioshiny).

The interconnections among sources (left), countries (middle), and author keywords (right) are analyzed to understand which keywords are preferable to which countries and used to what sources. The three top countries (Brazil, Turkey, and Spain) have a strong connection with the source ‘Food and Chemical Toxicology’ and prefer to publish four keywords (*Drosophila melanogaster*, genotoxicity, SMART, and antigenotoxicity). The block length presented in Fig. 6 indicates the level of connection.

#### Document co-citation and citation bursts

**Fig. 7.**
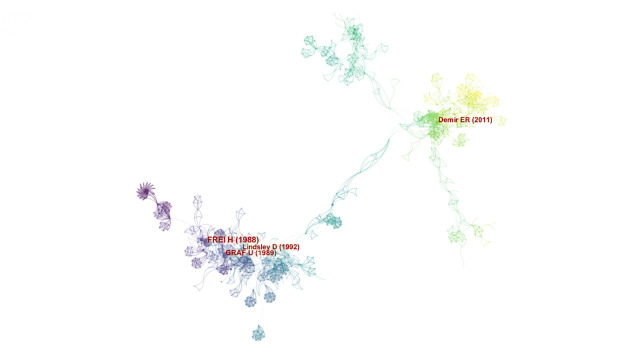
Co-citation network of SMART assays field. Each link colors indicate a given time slice. The oldest co-citation relationships are visualized as dark blue, whereas yellow links presented articles that are recently co-cited (created by CiteSpace). SMART, somatic mutation and recombination test.

**Fig. 8.**
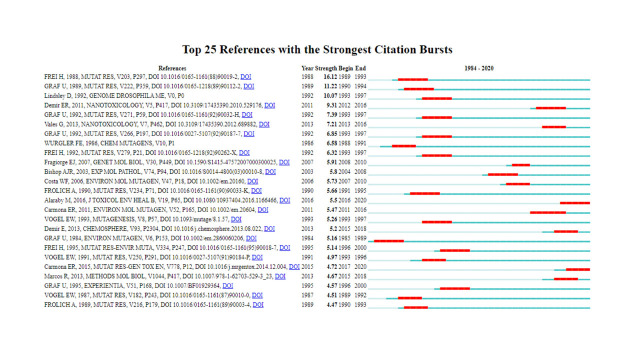
The top 25 references of somatic mutation and recombination test assays literature. The blue lines represent the time span (1984‒2020), the red lines denote the period of the bursts, strength indicates the burst strength (created by CiteSpace).

A co-citation network was generated with 985 nodes and 3,291 links for a one-year time slice (Fig. 7). The cited references are representing in the form of nodes and the co-citation relationships are visualized in the form of links. The top five co-cited articles are shown in Table 5 [14,41,47-49]. From a total of 392 records and 16,734 references, a list of the top 25 references with the strongest citation bursts was generated (Fig. 8). The citation bursts in the list of the top 25 references have been expanded between 1989 and 2017. Most of the strength bursts ranged between 4 and 7, and most citations have 3 to 4 years expanded duration. However, the citation with the most expanded duration (2011‒2016) has a low strength of its citation bursts (5.47) [50]. The reference paper written by Frei and Wurgler (1988) had the highest citation burst (16.12) [47].

### Thematic analysis

**Fig. 9.**
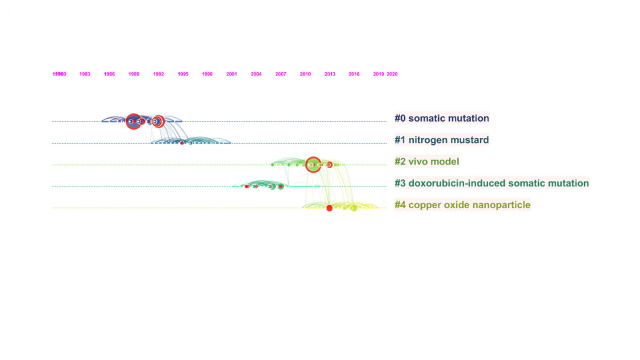
Timeline view of the largest five clusters of document co-citation based on one-year time slices from 1984 to 2020. The order of cluster from top to down is related to their size, and the arrangement of clusters from left to right denotes their time distribution. The frequency of co-citations is proportional to the size of nodes. The red rings around the nodes represent the citation bursts (created by CiteSpace).

A total of 17 clusters were identified in the SMART assays literature. The largest clusters are shown in Fig. 9. Based on specific metrics, term frequency‒inverse document frequency, log-likelihood tests (LLR), and mutual information tests, CiteSpace analyses the title of articles to extract a noun to characterize the cluster type. Generally, LLR covers the best themes (Table 6). A silhouette value (S) >0.7 denotes the high credibility of a cluster and a value of modularity (Q) >0.3 reveals the significant structure of the network [51]. As shown in Figs. 7 and 8, the top-ranked item by centrality is Frei and Wurgler (1995) [40] in Cluster #2, with the centrality of 41. The second one is Carmona et al. (2011) [50] in Cluster #3, with the centrality of 38. The third is Demir et al. (2011) [49] in Cluster #2, with the centrality of 34. Therefore, these papers are considered as pivotal points that allow connections between the research area. The clusters #0, #2, and #3 are the most active clusters with the strongest citation bursts (Table 7) [14,4,48-50,52-55]. This implies that these clusters denote where the supreme effort of research in the SMART assay.

## Discussion

In the current study, a comprehensive analysis of the emerging trends in the field of SMART assays from 1984 to 2020 was performed. The analysis reveals an increase in the number of publications in this period, with most of these having been published in the last 10 years, confirming the growing interest in SMART assays. Of note, recently published studies have received fewer citations compared to ancient studies, as it requires time for a study to make an impact. The first observed bloom of TLCS and TGCS has been associated with the first paper introducing the SMART assays in 1984. Thus, the highly cited article is the earliest publication. The second bloom in 1992 has been generated from two influential articles written by Graf and van Schaik (1992) [41] and Frei et al. (1992) [42]. The most prolific author in the SMARTs field is Graf U (Switzerland), with 953 TLCS and 1,716 TGCS. Furthermore, four of his research papers are in the top 10 highly cited articles, and five of them in the top 25 strongest citation bursts showing the interest of researchers in his field. The collaboration between authors could be due to the emergence of interest among researchers. While only one of the prominent clusters is interconnected, it is expected in the future to improve overall collaborative work. The large number of multi-authored documents could be related to various collaborations between countries to expedite the usage of SMART assays. In fact, 35 countries have published about SMART assays. The two influential authors de Andrade HHR and Lehmann M are both from Brazil. Thus, this country had the highest number of publications. The rise in publications from Brazil can also be attributed to the high frequency of collaboration with institutions in Switzerland. Both authors de Andrade HHR and Lehman M were collaborators with Graf U in 2000 to study the genotoxic potential of tannic acid [56]. However, the usage of SMART assays in genotoxicity studies is still ignored in many countries. The most influential journals accounted for 61.5% of all the publications, and this finding illustrated that the distribution of publication was narrow. These influential journals are in Q1(3), Q2(2), Q3(3), and Q4(2) category. To note, five highly cited articles were published in Mutation Research (Q1), two in Mutagenesis (Q2), and one in Environmental and Molecular Mutagenesis (Q2). The first most-cited article by Graf et al. was published in 1984 [11]. This article was published in Environmental Mutagenesis (Q2), ranked at 55th position with only this publication. Therein, the protocol of SMART assay was presented, and several chemicals such as β-propiolactone, 1,2-dibromoethane, aflatoxin B1, diethylnitrosamine, dimethylnitrosamine, mitomycin C, and procarbazine have been identified as mutagens. The second most-cited article by Frei and Wurgler [40] was published in Mutation Research-Environmental Mutagenesis and Related Subjects. This journal ceased publication, to be incorporated in 1997 into Mutation Research-Genetic Toxicology and Environmental Mutagenesis journal (Q3) [57]. In this study, to reduce the risk of inconclusive results, a new statistical test was proposed. The third most-cited article by Graf and van Schaik [41] proposed new strains to improve the visual score. This article was published in Mutation Research (Q1 in 1999) currently known as Mutation Research- Fundamental and Molecular Mechanisms of Mutagenesis (Q3) [57]. The most used keywords allow distinguishing the articles relevant to SMART assays. The three-fields plot showed that *Drosophila melanogaster*, genotoxicity, SMART, and antigenotoxicity were used by authors from Brazil, Turkey, and Spain. These keywords appear to be generic; yet it was used frequently. Based on thematic analysis, five large clusters were observed. The two clusters ‘somatic mutation’ and ‘nitrogen mustard’ are the two largest and oldest clusters. The cluster ‘copper oxide nanoparticle’ is the youngest cluster. The value of the mean silhouette (S) and the modularity (Q) are 0.9501 and 0.3944, respectively, suggesting reliable and robust results. In the largest cluster labeled ‘somatic mutation’ (#0) with 103 members and which is also the most active cluster, the most actively citing article (a research frontier article) identified polycyclic aromatic hydrocarbons and their derivatives as genotoxic [58]. The most actively cited articles (intellectual-based papers) are Graf et al. (1989) [14], Lindsley and Zimm (1992) [48], and Graf and van Schaik (1992) [41]. Graf et al. [14] is the second top-ranked paper with the strongest citation burst (11.22) and therein the efficiency of the SMART assay was proven by applying it on 30 compounds to evaluate their genotoxic potential. Lindsley and Zimm [48] described and identified the genome of *Drosophila*. Graf and van Schaik [41] improved new strain to assure better cross. In brief, cluster #0 mainly concentrated on enhancing the SMART protocol to be more practical and accurate. In the second cluster labeled ‘nitrogen mustard’ (#1), the most actively citing paper focused on the effect of tannic acid on nitrogen mustard, mitomycin C, and methylmehanesulfonate [56], whereas the most actively cited paper are Frei and Wurgler [40] and Graf [59]. These two papers focused on the *Drosophila* model by identifying the sample size required as well as the appropriate age of larvae, depending on the mutagens to avoid inconclusive results. It can be concluded that cluster #1 focused on the incorporation of a new control positive (nitrogen mustard) into SMART assay. Not surprisingly that the two largest clusters cover the most interests as both focused on the improvement of SMART assays protocol. In the third cluster labeled ‘vivo model’ (#2) the most actively citing paper was written by Carmona et al. [60] to evaluate the genotoxic potential of titanium dioxide anatase nanoparticles. Demir et al. [49] and Vales et al. [52] are the most-cited paper. Demir et al. [49] analyzed the genotoxic potential of silver nanoparticles whereas Vales et al. [52] evaluated the genotoxic effect of cobalt nanoparticles. It seems that in cluster #2 nanoparticles have gradually attracted the attention of scholars. Although the most-cited and citing papers were mainly dedicated to the genotoxic effect of nanoparticles, most of members treated various compounds, justifying thereby the label ‘vivo model’ instead of nanoparticles. In the fourth cluster labeled ‘doxorubicin-induced somatic mutation’ (#3), all the intellectual bases are related to cancer. Fragiorge et al. [53] analyzed the antigenotoxicity effect of ascorbic acid on doxorubicin. Doxorubicin is an antibiotic to treat human cancers which generally induces genotoxicity by oxidative damage [61]. Bishop and Schiestl [54] studied the function of homologous recombination in cancer. Similarly, the citing paper focused on the use of the herbal extract of ginseng to inhibit the genotoxic effect of doxorubicin [62]. In brief, cluster #3 was concentrated on resolving the doxorubicin-induced genotoxicity. The fifth cluster labeled ‘copper oxide nanoparticle’ (#4) is considered a persistent cluster denoting the continuity of an existing trend. In this cluster, the research frontier assessed the effect of copper oxide nanoparticles [63]. The metal oxide nanoparticles possess a redox property suggesting an antigenotoxic and anticarcinogenic potential [21]. The intellectual base emphasis on nanomaterials. Alaraby et al. [64] discussed the side effect of nanomaterials. Carmona et al. [60] presented the titanium dioxide anatase nanoparticles, being generally used in pharmaceuticals and cosmetics. This nanomaterial can generate oxidative stress, and subsequently genotoxicity [65]. Interestingly, this paper was a research frontier in cluster #2. It is worth mentioning, the first top-ranked burst item Frei and Wurgler (16.12) [47] is in a small and old cluster (1986) labeled ‘antiparasitic nitrofuran’ (#6) with 45 members. In sum, the research frontiers share sometimes the same theme with the intellectual bases since most often the continuation and the growth of intellectual base are the research fronts. The present study has certain limitations. Data were extracted from the WOS database; other databases were not considered. The database WOS is always updating, even with recently published papers, top publications are high enough that including recent paper would not have an influence on this research. The proceedings papers, book chapters, and early access are not included. Even though book chapters were excluded, the book by Wurgler and Vogel (1986) [12] describing the eye-spot assay was not indexed in the WOS database. The researchers tried carefully to include a maximum of relevant keywords; however, two studies Martinez-Valdivieso et al. (2017) [66] and Fernandez-Bedmar and Alonso-Moraga (2016) [67] have been missed. The absence of these papers is due to keyword search where none of the relevant used keywords were present in the title, neither in the abstract and/or the keywords section. The number of authors may differ since some authors published articles with a different initial of the first name. This led to some authors being separated into two authors. Even with these limitations, the current study on SMART assays can provide a directive to researchers to find influential articles, journals, and authors to assure collaboration and to reduce the research gaps.

In this study, a comprehensive bibliometric analysis was performed on SMART assays literature. Most of the publications were published in the last 10 years, mainly from Brazil, especially from the Federal University of Uberlândia. Notably, multiple authors were publishing papers on this field and mostly in ‘Food and Chemical Toxicology’ and ‘Mutation Research Genetic Toxicology and Environmental Mutagenesis’ journals. By analyzing the emerging trends, the nanoparticle area represents a persistent cluster. This area is expected to draw more attention. Finally, furthers studies should assess the records present on other databases to find out if the same trends for SMART assays are present.

## Figures and Tables

**Table 1.** The top 10 impactful authors ranked by Recs

Author	Recs	TLCS	TLCS/t	TGCS	TGCS/t
Graf U	42	953	33.47	1,716	65.59
Marcos R	39	235	21.23	775	79.41
de Andrade HHR	34	176	11.25	494	35.34
Lehmann M	33	113	8.93	327	29.67
Kaya B	30	163	14.85	449	41.8
Dihl RR	26	72	6.82	198	21.72
Creus A	25	199	15.39	603	51.26
Reguly ML	25	172	10.43	419	25.68
Spano MA	25	105	9.57	301	33.59
Demir E	23	99	10.91	276	35.09

Recs, number of publications; TLCS, total local citation score; TLCS/t, total local citation score per year; TGCS, total global citation score; TGCS/t, total global citation score per year.

**Table 2.** The most productive institutions and countries ranked by Recs

No.	Country	Institution	Recs	TLCS	TGCS	No.	Country	Recs	TLCS	TGCS
1	Brazil	Univ Fed Uberlândia	39	156	425	1	Brazil	106	361	1,103
2	Spain	Univ Autonoma Barcelona	38	227	763	2	Spain	69	338	1,470
3	Turkey	Akdeniz Univ	34	170	511	3	Turkey	66	221	784
4	Switzerland	Univ Zürich	30	1,119	1,892	4	Switzerland	41	446	1,016
5	Switzerland	Swiss Fed Inst Technol	27	347	770	5	Mexico	39	95	457
6	Brazil	Univ Fed Goias	24	47	260	6	Japan	30	72	405
7	Brazil	Univ Luterana Brasil	22	65	285	7	USA	25	489	923
8	Spain	Univ Cordoba	21	71	517	8	Greece	14	24	264
9	Brazil	Univ Fed Rio Grande do Sul	21	95	304	9	India	10	23	100
10	Mexico	Univ Nacl Autonoma Mexico	19	27	178	10	UK	10	91	179

Recs, number of publications; TLCS, total local citation score; TGCS, total global citation score.

**Table 3.** The most productive journals ranked by Recs

Journal	Recs	TLCS	TLCS/t	TGCS	TGCS/t	IF (2020)
*Food and Chemical Toxicology*	53	165	15.8	714	69.78	6.025 (Q1)
*Mutation Research-Genetic Toxicology and Environmental Mutagenesis*	53	250	17.55	1,085	81.73	2.873 (Q3)
*Environmental and Molecular Mutagenesis*	31	213	11.24	511	29.03	3.216 (Q2)
*Mutation Research*	30	610	20.28	1,128	37.78	2.11 (Q1)^a^
*Mutagenesis*	20	187	7.99	440	18.44	3.000 (Q2)
*Fresenius Environmental Bulletin*	17	34	3.35	53	5.78	0.489 (Q4)
*Chemosphere*	10	30	3.12	177	19.19	7.086 (Q1)
*Genetics and Molecular Biology*	9	28	2.2	63	6.01	1.771 (Q3)
*Mutation Research-Fundamental and Molecular Mechanisms of Mutagenesis* ^b^	9	28	1.12	114	5.78	2.433 (Q3)
*Toxicology and Industrial Health*	9	17	2.85	68	11.73	2.273 (Q4)

Recs, number of publications; TLCS, total local citation score; TLCS/t, total local citation score per year; TGCS, total global citation score; TGCS/t, total global citation score per year; IF, impact factor; Q, quartile.

a Data from 1999;

b Previously known as Mutation Research.

**Table 4.** The top 10 highly cited articles ranked by LCS

Title	Author	Source	Year	LCS	GCS
Somatic mutation and recombination test in *Drosophila melanogaster*	Graf et al. [11]	Environmental Mutagenesis	1984	319	527
Optimal experimental-design and sample-size for the statistical evaluation of data from somatic mutation and recombination tests (SMART) in *Drosophila*	Frei and Wurgler [40]	Mutation Research-Environmental Mutagenesis and Related Subjects	1995	133	156
Improved high bioactivation cross for the wing somatic mutation and recombination test in *Drosophila melanogaster*	Graf and van Schaik [41]	Mutation Research	1992	132	159
30 Compounds tested in the *Drosophila* wing spot-test	Graf et al. [14]	Mutation Research	1989	121	165
The genotoxicity of the anticancer drug mitoxantrone in somatic and germ-cells of *Drosophila melanogaster*	Frei et al. [42]	Mutation Research	1992	85	100
Induction of somatic mutation and recombination by four inhibitors of eukaryotic topoisomerases assayed in the wing spot test of *Drosophila melanogaster*	Frei and Wurgler [43]	Mutagenesis	1996	42	56
Metabolism of promutagens catalyzed by *Drosophila melanogaster* Cyp6a2 enzyme in *Saccharomyces cerevisiae*	Saner et al. [44]	Environmental and Molecular Mutagenesis	1996	41	91
Genotoxicity testing of antiparasitic nitrofurans in the *Drosophila* wing somatic mutation and recombination test	Moraga and Graf [45]	Mutagenesis	1989	37	47
*Drosophila* wing-spot test - improved detectability of genotoxicity of polycyclic aromatic-hydrocarbons	Frolich and Wurgler [46]	Mutation Research	1990	29	50

LCS, local citation score; GCS, global citation score.

**Table 5.** The top five critical articles in SMART assays

Cited frequency	Title	Author	Year	Betweenness centrality
30	Statistical methods to decide whether mutagenicity test data from *Drosophila* assays indicate a positive, negative, or inconclusive result	Frei and Wurgler [47]	1988	0.08
21	30 Compounds tested in the *Drosophila* wing spot-test	Graf et al. [14]	1989	0.13
19	The genome of *Drosophila melanogaster*	Lindsley and Zimm [48]	1992	0.08
19	Genotoxic analysis of silver nanoparticles in *Drosophila*	Demir et al. [49]	2011	0.12
14	Improved high bioactivation cross for the wing somatic mutation and recombination test in *Drosophila melanogaster*	Graf and van Schaik [41]	1992	0.12

SMART, somatic mutation and recombination test.

**Table 6.** The top five clusters in the literature

ID	Size	Silhouette	Label (TF-IDF)	Label (LLR)	Label (MI)	Cited year
0	103	0.910	*Drosophila melanogaster*	Somatic mutation	Inhibitory activity	1989
1	97	0.864	*Drosophila melanogaster*	Nitrogen mustard	Pyrrolizidine alkaloid	1995
2	79	0.932	*Drosophila melanogaster*	Vivo model	Grifola gargal singer	2010
3	77	0.961	*Drosophila melanogaster*	Doxorubicin-induced somatic mutation	Sage tea	2005
4	74	0.977	*Drosophila melanogaster*	Copper oxide nanoparticle	Grifola gargal singer	2015

TF-IDF, term frequency‒inverse document frequency; LLR, log-likelihood tests; MI, .

**Table 7.** The top three articles in clusters #0, #2, and #3 with the strongest citation bursts

ID	Burst	Title	Author	Year
0	11.22	30 Compounds tested in the *Drosophila* wing spot-test	Graf et al. [14]	1989
	10.07	The genome of *Drosophila melanogaster*	Lindsley and Zimm [48]	1992
	7.39	Improved high bioactivation cross for the wing somatic mutation and recombination test in *Drosophila melanogaster*	Graf and van Schaik [41]	1992
2	9.31	Genotoxic analysis of silver nanoparticles in *Drosophila*	Demir et al. [49]	2011
	7.21	Genotoxicity of cobalt nanoparticles and ions in *Drosophila*	Vales el al. [52]	2013
	5.47	Proposal of an *in vivo* comet assay using haemocytes of *Drosophila melanogaster*	Carmona et al. [50]	2011
3	5.91	Modulatory effects of the antioxidant ascorbic acid on the direct genotoxicity of doxorubicin in somatic cells of *Drosophila melanogaster*	Fragiorge et al. [53]	2007
	5.80	Role of homologous recombination in carcinogenesis	Bishop and Schiestl [54]	2003
	5.73	Protective effects of a mixture of antioxidant vitamins and minerals on the genotoxicity of doxorubicin in somatic cells of *Drosophila melanogaster*	Costa and Nepomuceno [55]	2006
